# Supplementary material for: A preliminary study into internet related addictions among adults with dyslexia
Source: PLoS One. 2023 Feb 24;18(2):e0280555. doi: 10.1371/journal.pone.0280555 (PMC9955639; doi:10.1371/journal.pone.0280555)
Supplement: Attachment — Submitted filename: A preliminary study into internet related addictions among adults with dyslexia_review_virag.docx [file pone.0280555.s002.docx]

Thank you for the opportunity to review the manuscript ‘A preliminary study into internet related addictions among adults with dyslexia’.

**General remarks**

With the growing population having learning difficulties and internet addictions, it is an important topic and a relevant research question whether these two phenomenon are related and if so, what is the possible mechanism behind that. The authors present a preliminary analysis of data investigating the differences between adults with dyslexia and controls in terms of internet addiction: GIA (general internet addiction), SMA (social media addiction) and IGD (internet gaming disorder). The authors found that adults with dyslexia had higher levels of GIA and IGD compared to controls, however previously hypothesized differences in social media addiction were not found between the two groups.

Although this is a very important topic, and the manuscript is very thorough in explaining the results, I have found some data missing about the participants, and some remarks concerning the introduction and the discussion.

**Remarks concerning the manuscript**

**Abstract** is a clear summary of the manuscript.

**Introduction**

Page 3: “There are several reasons to suspect that dyslexia might be associated with these types of additions.” The authors should further explain in the manuscript what they mean by several reasons.

Page 4, paragraph 2: The Authors explain how mental health issues as consequences of ASD and ADHD may lead to internet addictions. In order to do so, they line up articles about anxiety, depression and low self-esteem in children with ASD and ADHD, and depression and anxiety as antecedent factors for internet related addictions. Importantly, the Authors base their hypotheses on these associations, as they imply that there is a similar association between dyslexia and internet related addictions. A more thorough explanation of how learning disabilities and internet related addictions might be associated is necessary, especially that the current data focuses on adults and some of the literature is about children.

Page 4, paragraph 3: A thorough and well written explanation about how SMA and dyslexia might be associated is presented. This would be necessary in the previous paragraph as well.

Page 6, paragraph 1: “Yet coping strategies may help mitigate the challenges and therefore research is needed to identify if those with dyslexia are susceptible to SMA, in the same way that those with ADHD are.” The Authors do not show literature or research on the comparison between ADHD and dyslexia, thus I suggest to take this comparison out.

Page 7, present study: Addiction is twice spelled as ‘addition’, please correct.

**Methods**

Page 8, participants: Authors state that all participants, including participants with dyslexia have no active mental health issues, however the assumption that dyslexia is relatable to internet addiction lies on the fact that people with dyslexia have higher levels of anxiety and depression. Was this controlled in the Prolific survey platform, and if so, how?

Page 9, sociodemographic characteristics of participants: Please provide age of participants as well.

Page 9, sociodemographic characteristics of participants: Data is fitted according to marital status, income, education and employment, however gender is not balanced, as male participants are almost double (n=186) compared to female (n=100). If this is a general sociodemographic ratio, it would be important to mention this in the introduction and how it might effect the association between learning disabilities, mental health issues and internet addiction.

Page 11: Suggestion to use ‘Analyses’ instead of ‘Analytical strategies’ as subtitle.

**Results**

Page12, Descriptive statistics and data screening: Descriptive statistics show that both dyslexia and control group fall into the ‘mild’ IA category, and neither group falls into the pathological category in either IGD or SMA. This is problematic, because in later phases of the manuscript, Authors state that dyslexia is related to IGD and IA, however IA is only mild for both groups, and IGD doesn’t reach pathological levels in neither of the two groups.

The authors imply that the dyslexia group shows higher results in all three scales, however with the standard deviations in mind, the two groups are highly overlapping, differences are only statistically significant after square root transformations, which is explained later. These significant differences don’t imply that participants with dyslexia have IGD.

Other than the comment above, results are clearly written and well explained.

Page 13, line 15: please correct ‘sccore’ to score

**Discussion**

Authors explain possible limitations of the study: its cross-sectional nature, and the lack of mental health questionnaires, and also make valid associations between current results and previous literature.

Page 19 paragraph 2: it is not clear from the manuscript what the Authors mean by ‘hidden problem’ particularly for people with learning disabilities. Please explain this a bit more in the introduction and the discussion of the manuscript.

Page 20, paragraph 2: IGD scores are higher for participants with dyslexia, however concerning the level of scores on the scales, it seems slightly far fetched to state that it is related to an actual addiction.

Page 20, paragraph 2: “Hence further attention is warranted because if significant relationships between dyslexia and GIA, SMA and IGD are detected early, then interventions can be undertaken to manage such problems for this group.”. Importantly, this preliminary study SMA was not higher for participants with dyslexia, therefore it is suggested to exclude it from this assumption.
